# An observational analysis of the impact of indoor residual spraying with non-pyrethroid insecticides on the incidence of malaria in Ségou Region, Mali: 2012–2015

**DOI:** 10.1186/s12936-017-2168-2

**Published:** 2018-01-10

**Authors:** Joseph Wagman, Christelle Gogue, Kenzie Tynuv, Jules Mihigo, Elie Bankineza, Mamadou Bah, Diadier Diallo, Andrew Saibu, Jason H. Richardson, Diakalkia Kone, Seydou Fomba, Jeff Bernson, Richard Steketee, Laurence Slutsker, Molly Robertson

**Affiliations:** 1 PATH, Washington, DC, USA; 2 PMI, Bamako, Mali; 3 Abt Associates, Bamako, Mali; 4 MEASURE Evaluation, Bamako, Mali; 5 Abt Associates, Accra, Ghana; 6 IVCC, Washington, DC, USA; 7 Programme National de Lutte Contre le Paludisme, Bamako, Mali; 8 PATH, Nairobi, Kenya; 9 PATH, Seattle, WA USA

**Keywords:** Indoor residual spraying, Malaria incidence, Observational analysis, Mali, Next generation IRS

## Abstract

**Background:**

Ségou Region in Central Mali is an area of high malaria burden with seasonal transmission, high access to and use of long-lasting insecticidal nets (LLINs), and resistance to pyrethroids and DDT well documented in *Anopheles gambiae* s.l. (the principal vector of malaria in Mali). Ségou has recently received indoor residual spraying (IRS) supported by Mali’s collaboration with the US President’s Malaria Initiative/Africa Indoor Residual Spraying programme. From 2012 to 2015, two different non-pyrethroid insecticides: bendiocarb in 2012 and 2013 and pirimiphos-methyl in 2014 and 2015, were used for IRS in two districts. This report summarizes the results of observational analyses carried out to assess the impact of these IRS campaigns on malaria incidence rates reported through local and district health systems before and after spraying.

**Methods:**

A series of retrospective time series analyses were performed on 1,382,202 rapid diagnostic test-confirmed cases of malaria reported by district routine health systems in Ségou Region from January 2012 to January 2016. Malaria testing, treatment, surveillance and reporting activities remained consistent across districts and years during the study period, as did LLIN access and use estimates as well as *An. gambiae* s.l. insecticide resistance patterns. Districts were stratified by IRS implementation status and all-age monthly incidence rates were calculated and compared across strata from 2012 to 2014. In 2015 a regional but variable scale-up of seasonal malaria chemoprevention complicated the region-wide analysis; however IRS operations were suspended in Bla District that year so a difference in differences approach was used to compare 2014 to 2015 changes in malaria incidence at the health facility level in children under 5-years-old from Bla relative to changes observed in Barouéli, where IRS operations were consistent.

**Results:**

During 2012–2014, rapid reductions in malaria incidence were observed during the 6 months following each IRS campaign, though most of the reduction in cases (70% of the total) was concentrated in the first 2 months after each campaign was completed. Compared to non-IRS districts, in which normal seasonal patterns of malaria incidence were observed, an estimated 286,745 total fewer cases of all-age malaria were observed in IRS districts. The total cost of IRS in Ségou was around 9.68 million USD, or roughly 33.75 USD per case averted. Further analysis suggests that the timing of the 2012–2014 IRS campaigns (spraying in July and August) was well positioned to maximize public health impact. Suspension of IRS in Bla District after the 2014 campaign resulted in a 70% increase in under-5-years-old malaria incidence rates from 2014 to 2015, significantly greater (p = 0.0003) than the change reported from Barouéli District, where incidence rates remained the same.

**Conclusions:**

From 2012 to 2015, the annual IRS campaigns in Ségou are associated with several hundred thousand fewer cases of malaria. This work supports the growing evidence that shows that IRS with non-pyrethroid insecticides is a wise public health investment in areas with documented pyrethroid resistance, high rates of LLIN coverage, and where house structures and population densities are appropriate. Additionally, this work highlights the utility of quality-assured and validated routine surveillance and well defined observational analyses to rapidly assess the impact of malaria control interventions in operational settings, helping to empower evidence-based decision making and to further grow the evidence base needed to better understand when and where to utilize new vector control tools as they become available.

**Electronic supplementary material:**

The online version of this article (10.1186/s12936-017-2168-2) contains supplementary material, which is available to authorized users.

## Background

Over the past 15 years global efforts to control and prevent malaria have produced some astounding results. Estimates indicate that between 2000 and 2015 worldwide malaria incidence has declined almost 40% in areas at risk (from 146 cases per 1000 people per annum to 91 cases per 1000 people per annum) and malaria mortality rates have decreased by almost 60% in areas at risk (from 47 deaths per 100,000 people per annum to 19 deaths per 100,000 people per annum) [1]. This is reflective of worldwide progress in malaria that has come about, in part, through implementation of a package of standard evidence-based interventions of which malaria vector control has been a central component [1, 2]. Indeed, Bhat and others recently estimated that 81% of all malaria cases averted from 2000 to 2015 could be attributed to successful vector control interventions, namely the large-scale distribution and use of insecticide-treated nets (including long-lasting insecticidal nets, or LLINs) and the indoor residual spraying (IRS) of insecticides inside homes [3]. Even in Mali, where malaria control efforts are challenged by complex and intense transmission cycles as well as political instability, IRS and LLINs are thought to have contributed (along with improved malaria case management) to decreasing trends in all cause child mortality observed over the last 10 years [2, 4, 5].

While vector control is recognized as an integral part of the present and future global malaria control and elimination effort [6–8], the continued success of currently available tools is threatened by the rapid emergence and spread of insecticide resistance in key mosquito populations [9, 10]. Particularly worrisome is resistance to pyrethroids, the most commonly used class of insecticide for malaria vector control (and currently the only class available on LLINs), which is now widespread and found throughout most of sub-Saharan Africa [1]. Accordingly, WHO and others have recognized an urgent need to develop next-generation, non-pyrethroid insecticide products in order to preserve our ability to utilize IRS and LLINs for malaria prevention [2, 10].

Despite this need, widespread adoption of new products for IRS is hampered by several complex factors among which are concerns about increased costs as well as gaps that exist in the evidence base needed to evaluate the impact of newer products in various malaria transmission settings and in combination with other malaria control interventions. Nonetheless, there are a few currently available pyrethroid alternatives recommended by WHO for IRS targeting malaria vectors. Two of the most widely available products include a wettable powder (WP) formulation of bendiocarb, a carbamate insecticide, and a micro-encapsulated formulation of pirimiphos-methyl (PM CS), an organophosphate insecticide [11], both of which have been used by the US President’s Malaria Initiative (PMI) Africa Indoor Residual Spraying (AIRS) Project in recent years [12, 13]. While the insecticidal efficacy of bendiocarb has been well established experimentally [14, 15], and there are indications that it can have a positive impact in reducing malaria transmission in areas with pyrethroid-resistant mosquitoes [16–19], significant concerns about poor residual efficacy [20–22] and emerging resistance [23, 24] have recently led to a shift in PMI/AIRS operations away from spraying bendiocarb and towards the use of PM, both throughout Africa in general [12] and in Mali specifically [25]. However, wider-spread adoption and scale-up of PM has been hampered by its higher cost. To help meet this challenge, the Next Generation IRS (NgenIRS) project has introduced a co-payment mechanism for PM as part of a broad market-shaping effort for third generation IRS (3GIRS) products that includes wide-ranging impact analyses. By definition, a 3GIRS product is one that is designed to be effective against pyrethroid-resistant mosquitoes and have an indoor residual efficacy of at least 6 months.

Here, an observational, retrospective (2012–2015) time-series analysis of the seasonal epidemiological impact of IRS with bendiocarb WP and PM CS in the Ségou Region (Ségou) of central Mali is presented to: (1) help further expand the evidence base needed to evaluate the public health impact and cost effectiveness of IRS campaigns utilizing non-pyrethroid, including 3GIRS products; and (2) help foster the use of quality-assured and validated routine malaria surveillance data for evidence-based decision making.

## Methods

### Study site and malaria landscape

**Fig. 1:**
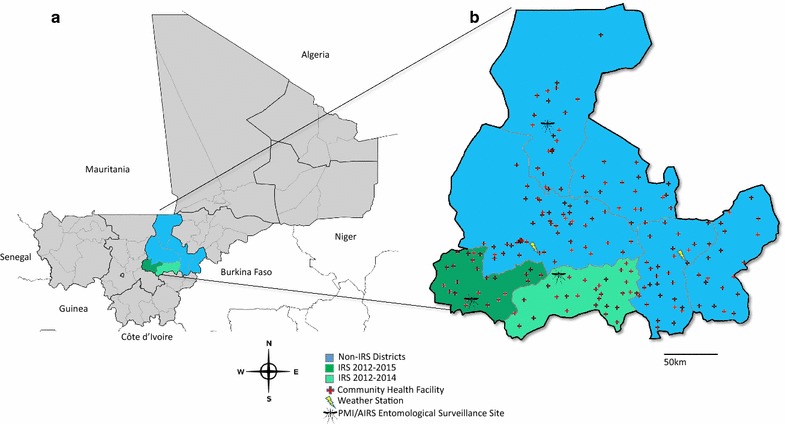
Study site. **a** The location of Mali in West Africa (left), with Ségou Region highlighted. **b** The locations of the community health facilities in Ségou that reported malaria RDT results during the months analysed here (Jan 2012 to Jan 2016). The IRS districts of Barouéli and Bla are shown in dark and light green, respectively; non-IRS comparator districts are shown in blue

Figure 1a illustrates the location of Ségou in the Niger River Valley of central Mali. The region is comprised of 7 administrative districts (*cercles*) covering roughly 65,000 sq km, with an estimated population (2013) of around 3 million—representing about 17% of the total population of Mali [26]. Contextually, Ségou has a relatively high malaria burden (56% prevalence in the under 5-years-old population (u5) according to the 2012/2013 Demographic and Health Survey [27]) with seasonal transmission patterns that are highest during and immediately after the rainy season, typically from June through September, when *Anopheles gambiae* sensu stricto (s.s.), *Anopheles coluzzii*, and *Anopheles arabiensis* are all present [28]. During the timeframe analysed here, Ségou reported consistently high metrics for LLIN access (86.5–90.1% of households owned at least one LLIN) and use (66.9–78.1% of children under 5 years old with access to a net slept under it the night preceding a survey) [27, 29, 30], local vector populations that were resistant to pyrethroids and DDT but susceptible to carbamates and organophosphates [28, 31], and a gradual scale-up of seasonal malaria chemoprevention (SMC) that began in 2013 [4]. Importantly, Ségou also contains two districts, Bla and Barouéli, selected to participate in the PMI/AIRS Project during this time, having been sprayed with bendiocarb WP in 2012 and 2013 and PM CS in 2014. In 2015, when SMC was expanded to include each district of Ségou, IRS operations were suspended in Bla but continued with PM CS in Barouéli (Table 1). Unless otherwise stated, years in which a district received SMC were excluded from the present analysis so that the impact of IRS on malaria transmission could be evaluated independently from of the impact of SMC.

**Table 1:** Summary of the malaria control landscape in Ségou Region, Mali 2012–2015

District	2012				2013				2014				2015			
	IRS				IRS				IRS				IRS			
	AI	Acceptance rate (%)^a^	LLINs distributed^b^	SMC^c^	AI	Acceptance rate (%)^a^	LLINs distributed^b^	SMC^c^	AI	Acceptance rate (%)^a^	LLINs distributed^b^	SMC (%)^c^	AI	Acceptance rate (%)^a^	LLINs distributed^b^	SMC (%)^c^
Barouéli	CA	98	Universal	–	CA	98	ANC/EPI	–	OP	96	ANC/EPI	–	OP	98	Universal	86
Bla	CA	99	Universal	–	CA	98	ANC/EPI	–	OP	98	ANC/EPI	104	None	–	Universal	98
Macina	None	–	Universal	–	None	–	ANC/EPI	–	None	–	ANC/EPI	–	None	–	Universal	82
Segou	None	–	Universal	–	None	–	ANC/EPI	–	None	–	ANC/EPI	–	None	–	Universal	82
Niono	None	–	Universal	–	None	–	ANC/EPI	–	None	–	ANC/EPI	–	None	–	Universal	68
San	None	–	Universal	–	None	–	ANC/EPI	94%	None	–	ANC/EPI	102	None	–	Universal	Unknown
Tominian	None	–	Universal	–	None	–	ANC/EPI	–	None	–	ANC/EPI	–	None	–	Universal	96

*AI* active ingredient, *CA* carbamate, *OP* organophosphate

^a^Percentage of structures targeted for IRS that were sprayed

^b^Universal distribution campaigns were undertaken in 2012 and 2015

^c^Percentage of target population (3–59 months of age) receiving at least 2 courses of SMC with SP + A

**Fig. 2:**
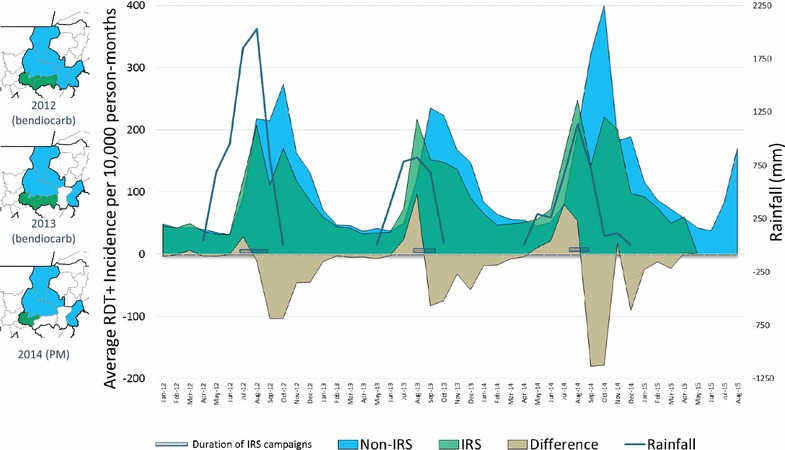
Reduction in malaria incidence following seasonal IRS campaigns in Ségou Region, Mali. Epidemiological curves showing the monthly incidence of RDT+ confirmed malaria cases in Ségou, stratified by district IRS status (inlayed maps). The area of the tan curve illustrates the difference between the incidence rates observed in the IRS districts (green) and the non-IRS districts (blue). The duration of each year’s IRS campaign is also illustrated

During the months analysed here, January 2012 to January 2016, the Système Numérique d’Information Sanitaire Intégré (SNISI) received a total of 9138 monthly malaria reports from 202 community health facilities (Centre de santé communautaires, CSCom) in Ségou (Fig. 1b). A total of 1,382,202 rapid diagnostic test (RDT)-confirmed cases of *Plasmodium falciparum* malaria were reported, with 81% of all cases being reported during June to January each year, corresponding to seasonal rainfall (Fig. 2). During this period, 90% of all suspected cases reporting to the health system in Ségou received an RDT and 69% of RDT-confirmed cases were correctly treated with an artemisinin combination therapy (ACT) [25]. No significant variation in these measures were observed across districts (ranges 90–98% tested; 65–78% treated) or years (ranges 92–97% tested; 68–72% treated). District reporting rates were greater than 98% for both IRS and non-IRS districts, although district-months in which no data were reported were excluded from analysis. Additionally, since 2012, MEASURE Evaluation has actively assisted the Ministry of Health with SNISI data quality assurance activities at all levels of the system, including tracking commodity stock-outs, which were minor during the period analysed here.

In addition to the availability of quality routine health facility data, Ségou provides an excellent setting for this impact analysis because the region includes two districts, Bla and Barouéli (Fig. 1), that implemented IRS with support from the PMI/AIRS programme. A third district, San, implemented SMC beginning in 2013, as did Bla (in addition to IRS) in 2014 and all remaining districts in 2015. The remaining four districts of Ségou (Macina, Ségou, Niono, and Tominian) received neither IRS nor SMC during the analysis period and are hereafter referred to as ‘non-IRS’ districts for comparison purposes. All districts benefitted from access to intermittent preventive treatment in pregnant women (IPTp) and high levels of LLIN access (92% in 2013) and use (75% in 2013) (Table 1) [27]. For the sake of this study, LLIN indicators are assumed to be similar across all districts of Ségou Region. Lastly, the districts of Ségou have also been shown to be similar to one another with respect to population density, rainfall patterns, malaria transmission seasonality, and population-adjusted *Plasmodium* prevalence rates [32], further strengthening the case to use the non-IRS districts as time- and climate-matched comparator districts for these observational analyses.

### IRS intervention

IRS was implemented each year during the months of July through September (Fig. 2) with support from the PMI/AIRS programme, which in Mali implements and blanket spraying strategy that targets all eligible houses in each spray district. In 2012 and 2013, houses in Barouéli and Bla districts were sprayed with bendiocarb WP (Ficam^®^; Bayer CropScience, Leverkusen, Germany); 2012 indicators show that 98.4% of targeted houses were sprayed in Barouéli (256,508 people protected, greater than 99% of the estimated district population) and 99.0% of targeted houses were sprayed in Bla (314,367 people protected, 95% of the district population) [33]; for 2013, 97.9% of targeted houses were sprayed in Barouéli (276,120 people protected, greater than 99% of the population) and 98.4% in Bla (346,675 people protected, greater than 99% of the population) [34]. In 2014, a switch in active ingredient was made and houses from both districts were sprayed with PM CS (Actellic^®^300CS, Syngenta AG, Basel, Switzerland). Spray coverage indicators were again similar in 2014, with 96.5% coverage of targeted houses in Barouéli (279,441 people protected, greater than 99% of the population) and 98.2% coverage in Bla (334,115 people protected, 95% of the population) [35]. In 2015, only Barouéli district was sprayed with PM CS, resulting in 98.3% coverage of targeted houses and 273,681 people protected (greater than 99% of the population) [36].

### Estimation of malaria incidence rates

Health facility catchment area and district population estimates, obtained from the *Ministère de la Santé de la République du Mali, Direction Régionale de la Santé*, were based on 2012 census results with an assumed simple linear growth function. Monthly malaria incidence rates were calculated by dividing the appropriate total number of RDT positive (RDT+) test results recorded in the SNISI database by the corresponding total district or health facility catchment area population denominators, as appropriate.

### Observational analysis of the impact of IRS on incidence rates: 2012–2014

For this analysis of monthly trends in all-ages malaria incidence, a quasi-experimental time series approach was used. Districts were stratified by IRS status (IRS and non-IRS), based on data compiled from PMI/AIRS end-of-spray reports and national Malaria Operational Plans [33–36]. Data from the health district of Markala were reported and analysed separately from data reported from Ségou District, but these two health districts are represented geographically as a single non-IRS district in the maps presented here. The total number of RDT+ test results reported were aggregated accordingly and epidemiological curves showing the incidence of RDT confirmed malaria cases reported per 10,000 person-months were plotted by calendar month for each IRS stratum.

To describe the seasonal impact of IRS, the cumulative incidence of RDT+ malaria cases observed during the 6 months following each IRS campaign (which also corresponds to the high transmission season) was calculated for the IRS districts and compared to the cumulative malaria incidence observed in the non-IRS districts during the same months. The total number of fewer cases observed during each 6-month, post-IRS window was calculated by multiplying the observed cumulative incidence reduction by the corresponding strata population estimates.

### Estimates of the cost-effectiveness of IRS

Reported IRS expenditure and costs were compiled from Revised PMI Obligation Tables [37] and the PMI IRS Country Programmes 2014 Comparative Cost Analysis [38]. Two crude estimates of the cost-effectiveness for each year’s IRS campaign were calculated by dividing either: (1) the total number of people protected based on the Ministry of Health population estimates or, (2) the total number of cases averted by the corresponding PMI/AIRS cost estimates for that year, adjusted for the proportional number of structures sprayed per district included in this analysis. For example, in 2013 42% of all the structures sprayed by PMI/AIRS in Mali were in Bla District; it was therefore assumed that operations in Bla represented 42% of the total IRS cost for that year. All costs are presented in adjusted 2014 USD.

### Follow-on analysis: modelling the possible impact of having shifted the IRS campaign start date

To explore possible effects that altering the IRS start dates could have had on the overall effectiveness of the campaigns in Ségou, a crude model describing the average monthly impact of each year’s IRS campaign was constructed. First, the monthly differences in observed malaria incidence between IRS and non-IRS districts across all 3 years were averaged to model the general monthly impact of IRS from 2012 to 2014, centred around ‘month zero’—the month in which IRS operations began (July in 2012 and 2014, August in 2013) (Additional file 1). Next, the observed monthly malaria incidence rates were averaged across all 3 years for both the IRS and non-IRS comparator districts, producing a single representative calendar year for each group (Additional file 1). The general impact model from step one was then applied to the average incidence rates from the non-IRS districts, maintaining the IRS start date in July; as expected, the resulting model curves were identical to the observed averages, providing some validation of the model approach (Additional file 1). Finally, the impact model was re-applied to the average non-IRS district incidence rates, sequentially shifting the modelled IRS campaign start date for each month from May to September.

### Estimating the impact of suspending IRS operations in Bla District, 2015

For the 2015 malaria transmission season, the regionally expanded (but variable) SMC coverage complicated the type of observational analysis described above, as there were no longer any appropriate non-IRS, non-SMC comparator districts for evaluation. Nonetheless, comparable 2015 SMC coverages achieved in Barouéli (85.76% coverage over four rounds) and Bla (97.75% coverage over three rounds) [39], coupled with the continuation of IRS operations in Barouéli, allowed for assessment of the impact of removing IRS operations from Bla that year. To explore this, a difference-in-differences approach was used to compare changes in malaria incidence observed at individual health facilities from year to year across both districts.

In short, monthly u5 malaria incidence rates were calculated for each health facility in Bla (26 facilities) and Barouéli (29 facilities). Differences in the cumulative u5 incidence from the 2014 transmission season to the 2015 transmission season were calculated and mapped. Lastly, the average change in incidence observed in the health facilities in Bla, which was sprayed in 2014 but not 2015, was compared to the average change in incidence observed at the health facilities in Barouéli, which was sprayed consistently in both years, using Student’s *t* test at α = 0.05. The difference-in-differences analysis presented here focused on trends in malaria incidence specific to the u5 population in order to standardize the impact of SMC across the districts, which was targeted to children ages 3–59 months, though all results and conclusions were the same in a subsequent sensitivity analyses using all-ages population incidence rates.

### Data analysis and visualization

Datasets were organized, cleaned, transformed, and joined using Microsoft Excel 2013 with Power Query v 2.41 (Microsoft Corp, Redmond, WA, USA) and Tableau v 10.0 (Tableau Software Inc, Seattle, WA, USA). Descriptive statistics were calculated using Excel 2013 and Tableau v10.0. Confidence intervals were calculated and Student’s t-tests comparing averaged incidence rates of malaria across districts by IRS status were conducting using STATA SE 14.2 (StataCorp, College Station, TX, USA). Geographical Information System analysis and mapping were performed using QGIS v2.16. Shapefiles were downloaded from the GADM database of Global Administrative Areas [40] in August, 2016. Entomological indicator datasets were compiled from the corresponding PMI/AIRS Entomological Surveillance Reports [28, 41, 42] and monthly rainfall summaries for Ségou region were compiled from free public datasets available through the Climate Data Online portal of the US National Oceanic and Atmospheric Administration, National Centers for Environmental Information [43]. Rainfall data presented are monthly averages of reports from two weather stations located in Ségou: Ségou (13.40N, 6.15E) and San (13.33N, 4.83E).

## Results

### Estimating the impact of IRS: 2012–2014

Epidemiological curves from January 2012 to August 2015 showing the average monthly incidence of RDT+ test results per 10,000 person-months, contrasting IRS only districts (Bla and Barouéli in 2012 and 2013; Barouéli only in 2014) with comparable non-IRS districts, are shown in Fig. 2. Each year during the 6 months of peak malaria transmission that followed IRS implementation, cumulative incidence was lower in the IRS districts than in non-IRS districts (illustrated by area of the tan curve in Fig. 2): in 2012, 2013 and 2014, 321, 290 and 492 fewer cases per 10,000 person-months, respectively, were observed after IRS. This equates to an estimated 108,604 total fewer malaria cases observed than would have otherwise been expected in 2012; 101,880 fewer total cases were observed in 2013; and 76,261 fewer total cases were observed in 2014 (when only one spray district was analysed) (Table 2).

**Table 2:** A crude estimate of the cost-effectiveness of IRS in Bla and Barouéli Districts, 2012–2014

Transmission season^a^	Reduced incidence observed in IRS districts (number of RDT+ cases per 10,000 person-months)	Total population of IRS districts	Estimated cases averted^b^	Estimated cost of IRS campaign^c^	Crude cost per person covered	Crude cost per case averted
2012	321.3	563,359	108,604	$3,426,834.00	$6.08	$31.55
2013	289.7	586,125	101,880	$4,339,328.78	$7.40	$42.59
2014	491.5	258,598	76,261	$1,778,893.77	$6.88	$23.33
Total			286,745	$9,545,056.55		

^a^Defined here as the 6 months following the IRS campaign

^b^(Reduced incidence) * (population of IRS districts) * (6 months)

^c^Annual Revised PMI Expenditures from Africa Indoor Residual Spray (AIRS) Mali Programme, in adjusted 2014 USD, multiplied by proportional number of houses sprayed per district analysed

### Crude cost effectiveness estimates for IRS

The populations of the districts that received IRS, the estimated total number of cases averted during the post-intervention periods, and the adjusted estimated costs of PMI/AIRS operations in those IRS districts are presented in Table 2. The crude cost of IRS was relatively consistent across years and across active ingredients, ranging from 6.06 to 7.40 USD per person protected and from 23.33 to 42.59 USD per case averted during peak transmission months (Table 2).

### Modelling the possible impact of having shifted the IRS campaign start date

**Fig. 3:**
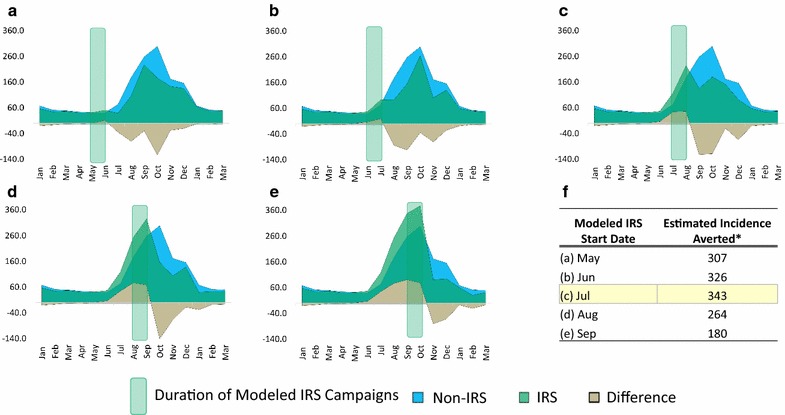
Model outputs showing potential impact of having changed the IRS campaign start date. The model estimates what the average impact of the IRS campaigns from 2012 to 2014 might have been if the campaign start dates had shifted **a** 2 months earlier, to May; **b** 1 month earlier, to June; **c** no shift, illustrating the actual impact of the campaigns that started in July; **d** 1 month later, to August; and **e** 2 months later, to September. The table shown in **f** shows the estimated number of cases per 10,000 person-months that were averted in each scenario, *based on the cumulative incidence from the 6 months after each modelled campaign

The initial epidemiological curves indicated that the timing of the IRS campaigns in 2012–2014 did not effectively target the first few weeks of increased malaria transmission that followed the beginning of the rainy season. To explore whether shifting the IRS start date could have improved overall campaign effectiveness, the IRS impact model was applied to five different start months: May (2 months earlier than the actual campaigns) through September (2 months later than the actual campaigns). Model outputs are summarized in Fig. 3. The graph in Fig. 3c shows the baseline model representing the actual campaign start dates, in which an estimated 343 cases per 10,000 person-months were averted over 6 months because of IRS (note that Fig. 3c also corresponds to the true averages of the real 2012–2014 IRS campaigns in Ségou). Shifting the start date 1 month earlier, to June, resulted in a small reduction of IRS impact: an estimated 326 cases per 10,000 person-months were averted (Fig. 3a). All of the other date shifts resulted in even further reductions of modelled IRS impact: starting in May (2 months earlier) averted around 300 cases per 10,000 person-months; starting in August (1 month later) averted around 260 cases per 10,000 person-months; starting in September (2 months later) averted only 180 cases per 10,000 person-months (Fig. 3).

### Epidemiological impact of the discontinuation of IRS in Bla District, 2015

**Fig. 4:**
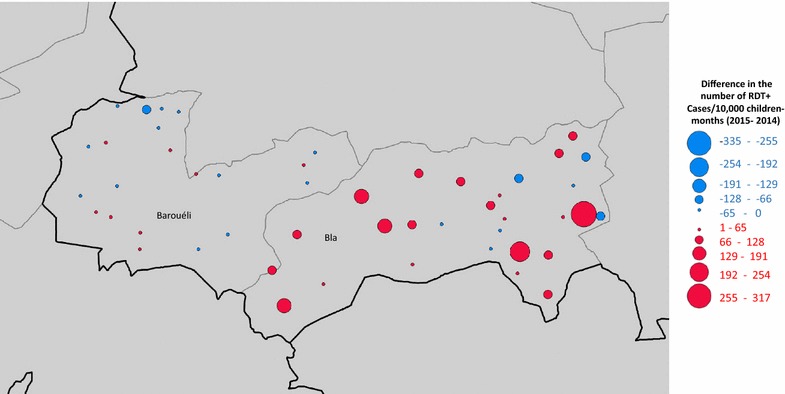
Changes in u5 malaria incidence (2014–2015) by health facility in Barouéli and Bla Districts. At each health facility location on the map, the colour blue represents a decrease in malaria rates from year to year while the colour red represents an increase in malaria rates. The magnitude of the change is represented by the size of the marker. Aggregated by district, malaria rates were the same from year to year in Barouéli, where IRS with operations were consistent, but increased by 202 cases/10,000 (1386 total extra cases) in Bla, where IRS operations were suspended after 2014

Prior to the 2015 transmission season, IRS operations were withdrawn from Bla but continued in Barouéli. The differences in u5 malaria incidence observed from 2014 to 2015 at each health facility from Bla (29 facilities) and Barouéli (26 facilities) are shown in Fig. 4. Malaria incidence rates were relatively consistent across years in Barouéli (an average decrease of 1.6%, CI_95_ − 15, + 12%), while malaria incidence rates increased by an average of 70% (CI_95_ 36, 105%) in Bla, a significant difference-in-differences effect of 72% (p = 0.0003: Student’s t-test). In Bla, this represents an extra 202 cases per 10,000 children-months (a total of 1386 u5 cases) in 2015 compared to 2014.

## Discussion

The success that has been achieved in reducing the global burden of malaria over the past 15 years is impressive, and should engender enthusiasm about the prospects for continued progress. Clearly though, the malaria control landscape is becoming increasingly complex and the need for new tools and further investment is becoming increasingly urgent. Presented here are results of a set of observational studies that show that IRS campaigns in the Ségou Region of central Mali likely prevented at least 280,000 cases of all-ages malaria from 2012 to 2014 at a cost of around 7.00 USD per person protected, with additional evidence that discontinuing IRS in Bla District in 2015 likely contributed to the sharp increase in the incidence of malaria observed that year. Collectively, this work supports both the public health utility and the cost-effectiveness of IRS with two different non-pyrethroid insecticides in communities with high rates of LLIN access and use as well as documented pyrethroid resistance.

Important limitations of this study include its observational nature and a lack of supportive datasets needed to control for potential confounding or co-variable factors or fundamental differences between intervention and non-intervention areas. Even though the current analysis is strengthened by the fact that the IRS and non-IRS districts of Ségou are similar ecologically and demographically and matched in time, specific limitations include the inability to adequately quantify the uncertainty around the proportion of reduced case numbers observed in IRS districts is attributable to the intervention, and not to chance or other factors. Additional limitations include reliance on malaria RDT results from patients who passively reported to the health system, which do not account for issues of patient access to health services that are likely to disproportionately affect more rural facilities, as well as the different geographical and temporal resolutions at which various data sets were available in Ségou. Although seasonal spray status and monthly malaria incidence rates were available at the health facility level, entomological, rainfall and SMC data were limited to the district and/or regional levels, preventing some more detailed analyses (e.g., multivariate regression, ANOVA, etc.) that could prove valuable in the future as various surveillance capacities grow and become more aligned. The basic impact model built to estimate the effect of changing the IRS campaign start date assumes that the magnitude of the monthly effect of IRS relative to the beginning of spray activities is independent of timing against the beginning of the rains (and therefore also independent of overall mosquito abundance), an assumption that is largely untested and likely to differ by active ingredient and target vector species. It is possible that IRS could be more or less effective as mosquito populations and environmental conditions fluctuate seasonally, though how this would impact overall disease transmission is uncertain. While this uncertainty is not likely to change the interpretation of the crude model presented here, future work could address these questions and help refine more detailed models of seasonal vector control impact.

Despite these limitations, the work presented here helps paint a picture of how IRS with non-pyrethroid insecticides has had a positive public health impact in central Mali by reducing malaria incidence rates, supporting similar positive results reported from transmission settings in Uganda [19, 44], Sudan [45], Tanzania [46], Zimbabwe [47], and Zambia [48], but contrasting less encouraging results from Benin [49] and The Gambia [50] that showed no benefit to IRS on top of existing interventions. It is clear (Fig. 2) that the IRS campaigns of 2012, 2013 and 2014 each had a sizable public health impact in Ségou by substantially reducing malaria transmission. While the incidence reductions observed after spraying bendiocarb WP in 2012 (320 fewer cases per 10,000 person-months) and 2013 (290 fewer cases per 10,000 person-months) were more similar than the reduction observed in 2014 after spraying PM CS (which resulted in 490 fewer cases per 10,000 person-months, around a 60% greater cumulative incidence reduction), it should be noted that malaria incidence was particularly high throughout Mali in 2014 and there is no indication of how well bendiocarb may have performed in this context. Any impact of having switched active ingredients is difficult to interpret given the inherent differences between the malaria transmission seasons of 2013 and 2014. Indeed, the crude impact modelling that was done here seems to indicate that the impact of the two chemicals was relatively similar in terms of their monthly per cent reductions in incidence compared to baseline (i.e., comparable unsprayed districts) by month: coefficients of variation were similar across years whether or not there was a shift in active ingredient (Additional file 2).

The overall effectiveness of IRS in Ségou is further evidenced by the observation that suspension of IRS operations in Bla District in 2015 was associated with a significant increase (70%) in malaria incidence that year, while no increase was observed in neighbouring Barouéli District, where IRS operations with Actellic were consistent across both years (Fig. 4). Interestingly, this increase in malaria transmission associated with the suspension of IRS from Bla is evident even though the district-wide SMC campaigns achieved high coverage there in both years as well as in Barouéli in 2015. In this context, and given recent indications that SMC campaigns can significantly reduce malaria incidence in western Africa [51–54], these results from Bla could suggest a compelling argument for the simultaneous implementation of IRS and SMC strategies in high burden areas with good LLIN coverage.

Also apparent in the epidemiological curves from Ségou is that the timing of the IRS campaigns, which began in late July and early August, did not effectively target the first few weeks of increased transmission following the very beginning of the rainy seasons, which began in May (Fig. 2). While the planning of IRS operations is an extraordinary complex decision (that in Mali was informed by concerns about the length of residual efficacy of bendiocarb [33, 41]), it is nonetheless intriguing to speculate whether moving the IRS campaign start dates to an earlier month could have helped reduce these early season cases and, therefore, prevented more malaria overall. However, the model described here does not support this, with the increased coverage during the beginning of the rainy season achieved by spraying earlier counter-balanced by the fact that the period of maximum IRS impact (40–47% reduction) lasted for only 2 months after the end of the spray campaigns; interestingly, this was consistent across all years, regardless of which IRS product was used (Additional files 1, 2). When spray campaigns ended in August, these 2 months of maximum impact (September and October) aligned with the 2 months of otherwise peak transmission, maximizing the effect of the intervention in terms of total cases of malaria prevented. Although the 2012–2014 campaigns in Ségou appear to have been timed well to achieve maximum impact, it is worth noting that the model described here suggests that even with less-than-perfect timing, IRS can still have a substantial, cost-effective impact on reducing malaria burden, at least when erring on the side of spraying too early as opposed to spraying too late.

The estimates of cost-effectiveness calculated here are basic, and meant only to give a rough idea about the financial implications of IRS campaigns that may require the use of newer, more expensive insecticides in the face of resistance concerns. It is nonetheless encouraging that these estimates indicate that spray campaigns with non-pyrethroid, including 3GIRS, products in central Mali were cost-effective investments, ranging from 6.08 to 7.40 USD per person protected and 23.33 to 42.59 USD per case averted, regardless of which active ingredient was used. It is also encouraging that these estimates did not fluctuate much, but it should be stressed that the target vectors in Mali were fully susceptible to both chemicals throughout the study period and changes in resistance to a product or chemical class will likely reduce its efficacy, and therefore its cost-effectiveness.

**Fig. 5:**
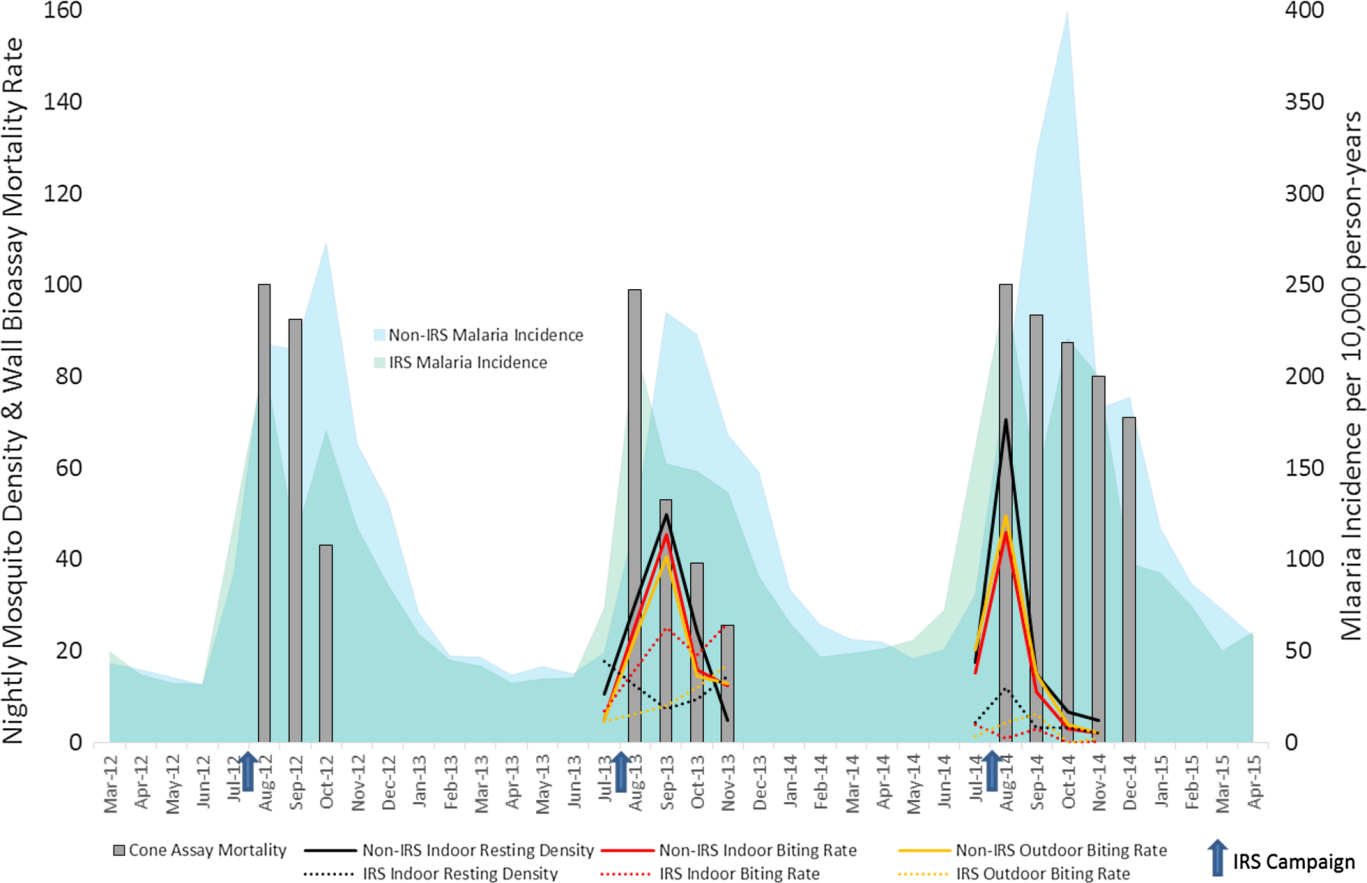
Comparing the entomological and epidemiological trends showing the impact of IRS in Ségou Region, 2012–2014. The monthly malaria incidence curves are presented with the results of PMI/AIRS entomological surveillance activities, including fluctuations in mosquito densities as measured by pyrethrum spray catches and human landing collections and the declining residual insecticidal efficacy as measured by standard WHO wall bioassay tests. Bendiocarb was sprayed in 2012 and 2013, while pirimiphos methyl was sprayed in 2014

It is also worthwhile to consider how these epidemiological trends fit with the entomological trends previously reported by PMI/AIRS [28, 33, 41, 42] (Fig. 5). Although the entomological surveillance datasets are of different spatial and temporal resolutions, and parallels with the incidence data should therefore be interpreted with caution, the PMI/AIRS reports have shown that IRS successfully controlled or reduced mosquito densities at IRS sentinel sites in Ségou, compared to the densities observed at the non-IRS sentinel site, results that support the epidemiological observations made here. The entomological trends held true for indoor resting mosquito densities collected via pyrethrum spray catch, which were 85% lower in IRS areas than control areas 2 months after the spray campaign in 2013 and 79% lower in 2014, and indoor biting rates calculated from human landing collections (HLCs), which were 45% lower in IRS areas than control areas 2 months after the spray campaign in 2013 and 73% lower in 2014 (Fig. 5). Interestingly, outdoor biting rates were also much lower at IRS sentinel sites than the control site, 80% lower after IRS in 2013 and 91% in 2014 (Fig. 5). This trend of higher malaria incidence rates corresponding to higher mosquito densities is also evident in Bla in 2015. After the cessation of IRS following the 2014 campaign, the 70% increase in malaria incidence observed in 2015 corresponds to a more than fivefold increase in peak (September) indoor HLC *An. gambiae* sensu lato (s.l.) densities (from 3 bites/person-night in 2014 to 2016 bites/person-night in 2015) (Additional file 3).

It is noteworthy that the waning residual efficacy of IRS, as measured using standard wall bioassays, corresponded to reduced entomological effect but not to reduced malaria prevention (Fig. 5). In 2013, the bendiocarb WP residual efficacy fell below the 80% wall test mortality threshold after just 1 month post-spray and mosquito densities returned to control levels by October, but the intervention appeared to contribute to a reduced malaria incidence for around 3 more months, until at least January (Fig. 5). After spraying PM in 2014, even though wall bioassay mortality stayed above 80% for a full 3 months and again maintained entomological control at the sentinel sites until the final collection, a similar duration of reduced malaria incidence was observed. There are many factors likely to influence these observations, among which are the natural lag time between the observed entomological and epidemiological effects of a malaria control intervention because of the external incubation period required, as well as the aforementioned fact that the disease surveillance data represent true district aggregates (cases reported from all health facilities located throughout an entire district) while the entomological surveillance, though robust, represents a much smaller geographical scale of just a few sentinel sites. Regardless, these observations highlight the need to develop better entomological indicators of potential epidemiological impact for various vector control interventions. Additionally, the data summarized here indicate that the residual efficacy of PM in central Mali was shorter than has been reported elsewhere [55–59], and additional assessments are underway to try to assess if regional climatic and/or housing material factors may influence product performance.

## Conclusions

Even when interpreted with appropriate caution, the observational analyses presented here indicate that the annual IRS campaigns from 2012 to 2015 in the Ségou Region of Mali were good public health investments. Around half a million people were protected for 3 years at a rough cost of around 7.00 USD or less per person per year regardless of which product was sprayed—similar to recent estimates of the cost of distributing LLINs to school children (around 5.50 USD per child [60]) or delivering community-based SMC (around 4.20 USD per child [54]) in Mali. These same IRS campaigns were also broadly associated in time and space with rapid decreases in malaria incidence and overall *An. gambiae* indoor resting and biting densities. It is encouraging that, despite the obvious need for an expanded IRS toolkit, campaigns with currently available non-pyrethroid products can still have a sizable, cost-effective impact in areas of high resistance and widespread use of LLINs. Nonetheless, the context of these analyses is relatively specific to central Mali, and further impact and cost-effectiveness studies in other regions and in other transmission settings would help enhance an understanding of how and when to maximize the impact of various vector control interventions.

Taken together, this work supports the growing evidence that shows that IRS with non-pyrethroid insecticides, including 3GIRS products, is a wise public health investment, especially in areas with documented pyrethroid resistance and high rates of LLIN coverage and where house structures and population densities are appropriate. Additionally, this work highlights the critical importance of quality-assured validated routine surveillance and well defined observational analyses to assess the impact of malaria control interventions in operational settings, helping to empower evidence-based decision making and to grow the evidence base needed to better understand when and where to utilize new vector control tools as they become available.

## Additional files

**Additional file 1.** The IRS campaign impact model as described in the text.

**Additional file 2.** The variation seen in IRS impact was similar between years and between active ingredients. CV: Coefficient of Variation. Also included are tabs showing the various model outputs showing the potential impact of having shifted the IRS campaign start dates.

**Additional file 3.** Increased malaria incidence corresponds to increased mosquito collection densities in Bla, 2014 to 2015. Indoor human landing collection (HLC) results overlaying the incidence curves. HLC results were not available for Barouéli in 2014, but the 2015 results are included for reference. The background curves show the monthly u5 malaria incidence rates from Bla (blue) and Barouéli (green).
