# Supplementary material for: An observational analysis of the impact of indoor residual spraying with non-pyrethroid insecticides on the incidence of malaria in Ségou Region, Mali: 2012–2015
Source: Malar J. 2018 Jan 10;17:19. doi: 10.1186/s12936-017-2168-2 (PMC5761159; doi:10.1186/s12936-017-2168-2)
Supplement: Supplementary file 3 — Additional file 3. Increased malaria incidence corresponds to increased mosquito collection densities in Bla, 2014 to 2015. Indoor human landing collection (HLC) results overlaying the incidence curves. HLC results were not available for Barouéli in 2014, but the 2015 results are included for reference. The background curves show the monthly u5 malaria incidence rates from Bla (blue) and Barouéli (green). [file 12936_2017_2168_MOESM3_ESM.pptx]

## Slide 1
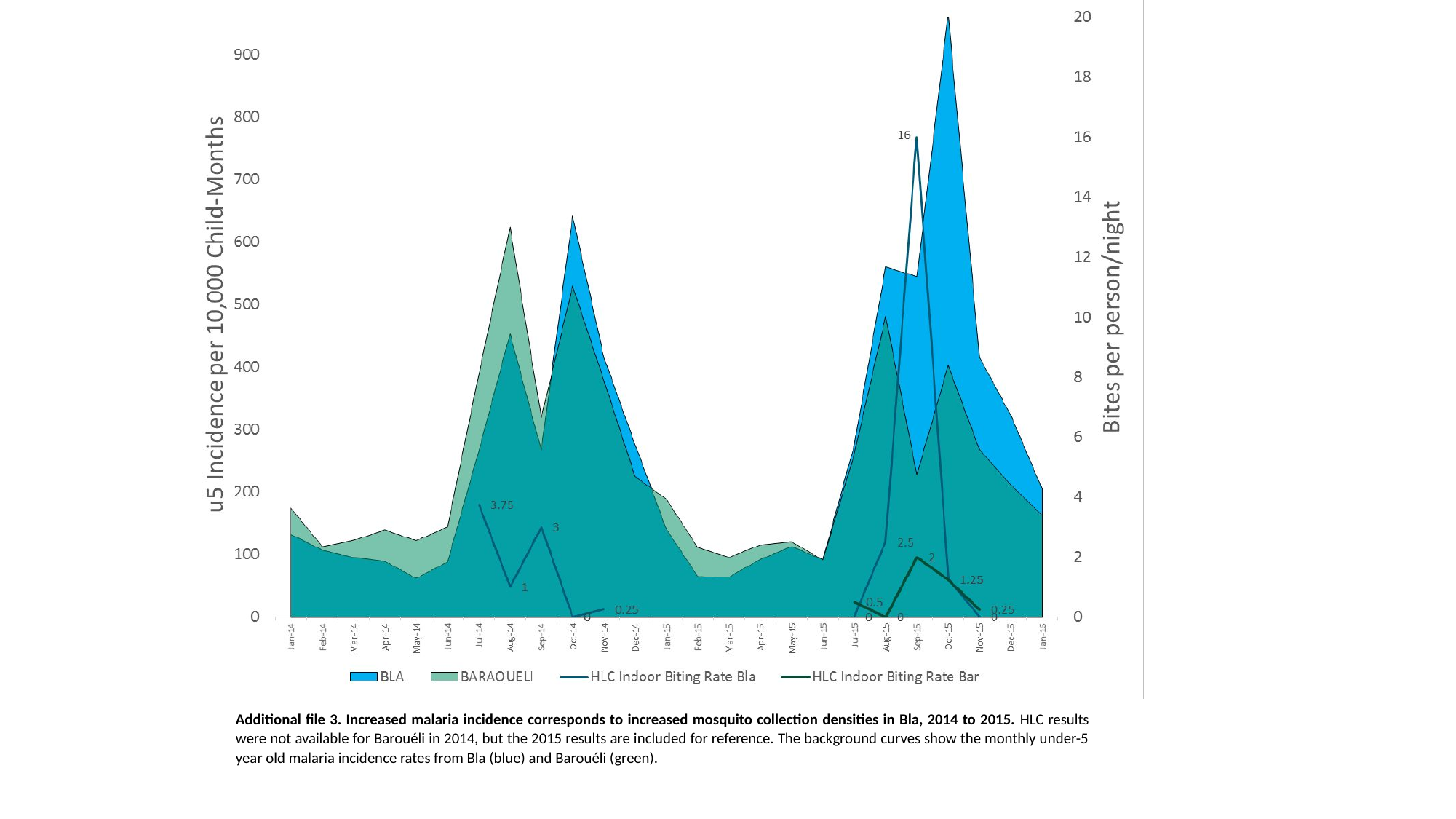

Additional file 3. Increased malaria incidence corresponds to increased mosquito collection densities in Bla, 2014 to 2015. HLC results were not available for Barouéli in 2014, but the 2015 results are included for reference. The background curves show the monthly under-5 year old malaria incidence rates from Bla (blue) and Barouéli (green).
